# Exercise-Induced Splanchnic Hypoperfusion Results in Gut Dysfunction in Healthy Men

**DOI:** 10.1371/journal.pone.0022366

**Published:** 2011-07-21

**Authors:** Kim van Wijck, Kaatje Lenaerts, Luc J. C. van Loon, Wilbert H. M. Peters, Wim A. Buurman, Cornelis H. C. Dejong

**Affiliations:** 1 Top Institute Food and Nutrition, Wageningen, The Netherlands; 2 NUTRIM School for Nutrition, Toxicology and Metabolism, Maastricht, The Netherlands; 3 Department of Surgery, Maastricht University Medical Center, Maastricht, The Netherlands; 4 Department of Human Movement Sciences, Maastricht University Medical Center, Maastricht, The Netherlands; 5 Department of Gastroenterology and Hepatology, Radboud University Nijmegen Medical Center, Nijmegen, The Netherlands; Technische Universität München, Germany

## Abstract

**Background:**

Splanchnic hypoperfusion is common in various pathophysiological conditions and often considered to lead to gut dysfunction. While it is known that physiological situations such as physical exercise also result in splanchnic hypoperfusion, the consequences of flow redistribution at the expense of abdominal organs remained to be determined. This study focuses on the effects of splanchnic hypoperfusion on the gut, and the relationship between hypoperfusion, intestinal injury and permeability during physical exercise in healthy men.

**Methods and Findings:**

Healthy men cycled for 60 minutes at 70% of maximum workload capacity. Splanchnic hypoperfusion was assessed using gastric tonometry. Blood, sampled every 10 minutes, was analyzed for enterocyte damage parameters (intestinal fatty acid binding protein (I-FABP) and ileal bile acid binding protein (I-BABP)). Changes in intestinal permeability were assessed using sugar probes. Furthermore, liver and renal parameters were assessed. Splanchnic perfusion rapidly decreased during exercise, reflected by increased gap_g-a_pCO_2_ from −0.85±0.15 to 0.85±0.42 kPa (p<0.001). Hypoperfusion increased plasma I-FABP (615±118 vs. 309±46 pg/ml, p<0.001) and I-BABP (14.30±2.20 vs. 5.06±1.27 ng/ml, p<0.001), and hypoperfusion correlated significantly with this small intestinal damage (r_S_ = 0.59; p<0.001). Last of all, plasma analysis revealed an increase in small intestinal permeability after exercise (p<0.001), which correlated with intestinal injury (r_S_ = 0.50; p<0.001). Liver parameters, but not renal parameters were elevated.

**Conclusions:**

Exercise-induced splanchnic hypoperfusion results in quantifiable small intestinal injury. Importantly, the extent of intestinal injury correlates with transiently increased small intestinal permeability, indicating gut barrier dysfunction in healthy individuals. These physiological observations increase our knowledge of splanchnic hypoperfusion sequelae, and may help to understand and prevent these phenomena in patients.

## Introduction

Splanchnic hypoperfusion occurs in many pathophysiological conditions, and is often considered to lead to epithelial barrier dysfunction. Vascular disease, trauma, and shock can induce splanchnic hypoperfusion and ischemia [1]–[2]. In addition, gastrointestinal (GI) dysfunction is one of the more frequent complications in surgical patients, in whom organ perfusion and oxygen delivery are often impaired as a consequence of surgery-induced alterations in cardio-respiratory and metabolic demands [3]. In critically ill patients, inadequate splanchnic blood flow causes intestinal damage, thereby compromising the intestinal mucosal barrier, potentially inducing and aggravating endotoxaemia and systemic inflammation [4].

Splanchnic hypoperfusion also occurs in physiological conditions. Young, healthy individuals endure episodes of splanchnic hypoperfusion during strenuous physical exercise [5], whereas older individuals may experience similar events during less exhausting activities. Moreover, there is considerable evidence supporting the theory that splanchnic hypoperfusion plays an important role in the development of GI complications in patients with chronic diseases, such as chronic heart failure or pulmonary disease [6]–[7]. Interestingly, the consequences of short-term splanchnic hypoperfusion on the organs in the splanchnic area remain to be determined.

In the current study, we focus on the direct effects of splanchnic hypoperfusion on gut, liver, and kidney epithelium in healthy young volunteers who perform moderate-to-high intensity physical exercise. This study is based on the fact that functional splanchnic hypoperfusion occurs during physical exercise, when rapid redistribution of the splanchnic blood flow occurs to secure supply of adequate amounts of oxygen and energy to the active muscle tissue, heart, and lungs [5], [8]. In addition, during exercise a reduction of the total circulatory blood volume, caused by transpiration and inadequate fluid intake, can decrease cardiac output and compromise splanchnic perfusion even more [9]. Especially during prolonged running or cycling, athletes can experience abdominal symptoms such as cramping, nausea, abdominal pain, and (bloody) diarrhea [10]. This points towards compromised GI functioning, but only few studies have looked at exercise-induced intestinal mucosal lesions in man [11]–[12].

In the current study, we determined the consequences of exercise-induced splanchnic hypoperfusion on gut, liver and kidney epithelium, with particular emphasis on intestinal barrier integrity loss in healthy individuals.

## Results

### Exercise-induced GI hypoperfusion

**Figure 1:**
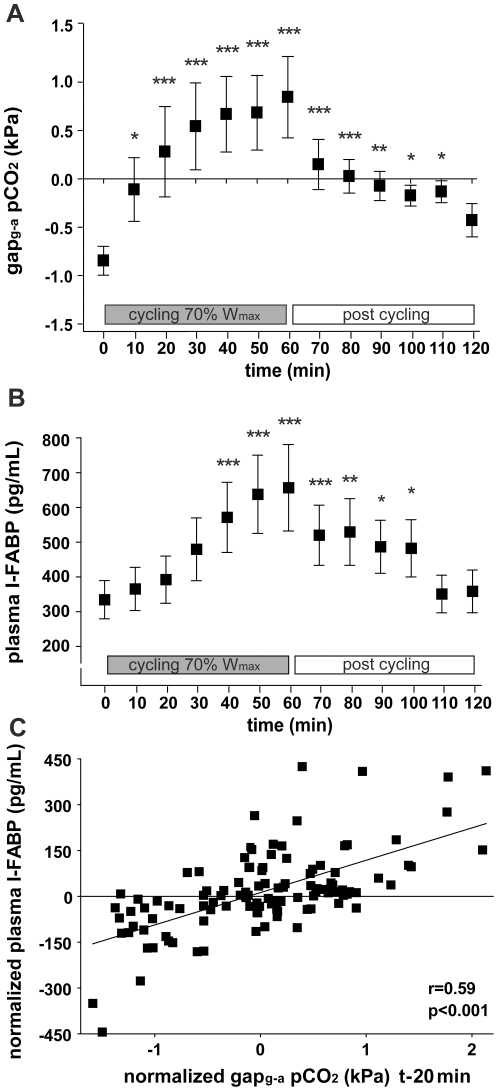
Physical exercise results in splanchnic hypoperfusion and intestinal cell damage. **A**) Gastric tonometry shows decreased splanchnic perfusion during cycling and post cycling (n = 9). **B**) Plasma I-FABP levels reflect the development of intestinal epithelial cell damage during cycling and post cycling in healthy volunteers (n = 20). **C**) Normalized plasma I-FABP levels measured during exercise tonometry correlate significantly with the normalized values of splanchnic hypoperfusion (gap_g-a_ pCO_2_ t-20 min) in healthy men (samples from 9 men). Data are mean ± SEM. Different from baseline (t = 0) (* p<0.01, ** p<0.001, *** p<0.0001).

Tonometry revealed a significant increase of gastric-arterialized pCO_2_ (gap_g-a_pCO_2_) during exercise, from −0.85±0.15 kPa to 0.85±0.42 kPa (p<0.001) at completion of exercise bout, indicating functional splanchnic hypoperfusion (Figure 1A; test design in Figure S1). The steepness of the gap_g-a_pCO_2_ slope was most pronounced during the first 10 minutes of exercise, suggesting that functional splanchnic adaptations occur rapidly after altered perfusion demands in other parts of the body during physical activity. The gap_g-a_pCO_2_ approximated baseline within 1 hour after exercise, reflecting fast recovery of splanchnic perfusion following a state of functional GI hypoperfusion. Interestingly, the recovery of GI perfusion was most prominent during the first 10 minutes post exercise, which is in line with the rapid adaptation of abdominal perfusion during the first 10 minutes of cycling.

### Exercise-induced small intestinal damage

To assess hypoperfusion-induced effects on the small intestinal epithelium, plasma intestinal fatty acid binding protein (I-FABP) levels were determined, which increased rapidly, from 309±46 pg/ml at baseline to 615±118 pg/ml (p<0.001) after cycling (Figure 1B). Similar to the decreased gap_g-a_pCO_2_ levels (Figure 1A), circulating I-FABP levels declined substantially in the first 10 minutes post exercise, and gradually decreased further until baseline I-FABP level was reached approximately 50 minutes after cycling (Figure 1B). Interestingly, we did not observe a new peak in plasma I-FABP levels in the 60 minutes after cycling, suggesting that reperfusion injury did not occur in this time period.

The short half-life of I-FABP in plasma (approximately 11 minutes) allows to analyze the correlation of enterocyte damage with splanchnic hypoperfusion [13], calculating the within-person correlation between gap_g-a_pCO_2_ and the change in circulating I-FABP. Because this correlation has not yet been described in healthy individuals, we determined the correlation between gap_g-a_pCO_2_ and I-FABP 3 times, i.e. with a delay of 10, 20, and 30 minutes. Interestingly, I-FABP levels significantly correlated with gap_g-a_pCO_2_ assessed at all measured time points (Spearman correlation coefficient (r_S_): 0.579 (p<0.001); 0.592 (p<0.001) and 0.528 (p<0.001), respectively). The correlation between gap_g-a_pCO_2_ and I-FABP levels determined in samples taken 20 minutes later, is depicted in Figure 1C.

**Figure 2:**
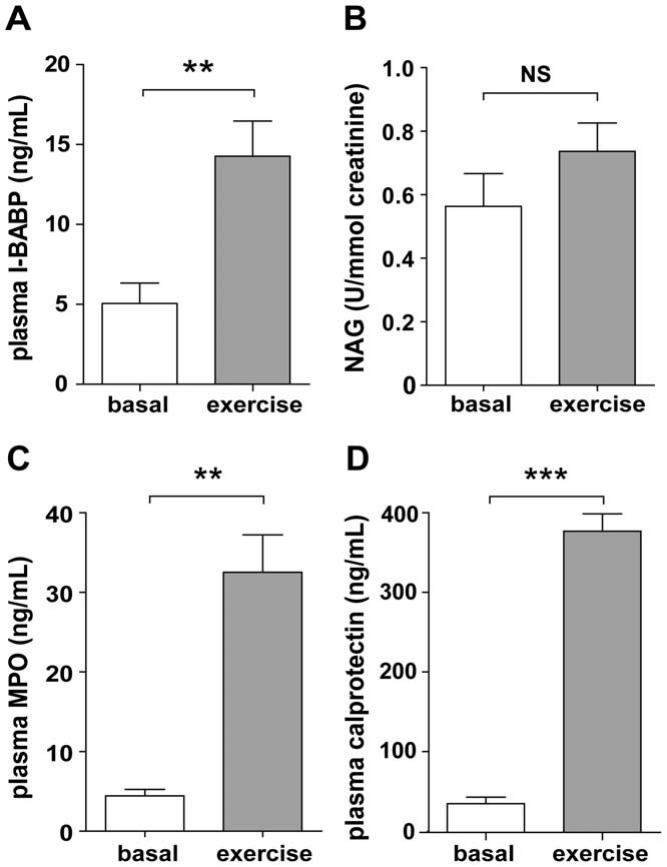
Physical exercise leads to ileal epithelial damage and inflammation, but does not result in renal injury. **A**) Increased plasma IBABP levels point toward the development of exercise-induced ileal epithelial cell damage. **B**) Urinary NAG levels indicate absence of renal damage after cycling. Increased plasma MPO (**C**) and calprotectin (**D**) levels reveal exercise-induced inflammation in healthy volunteers. Data are mean ± SEM (n = 20). Different from baseline (t = 0) (NS, not significant; ** p<0.001, *** p<0.0001).

Similar to I-FABP, cycling increased ileal bile acid binding protein (I-BABP) levels (5.06±1.27 to 14.3±2.20 ng/ml (p<0.001; Figure 2A)), reflecting enterocyte damage in the ileum specifically.

### Exercise-induced GI complaints

Two individuals reported nausea during the GI perfusion measurements with the nasogastric tube *in situ*, which resolved after the nasogastric tube was removed upon completion of the experiment. Three additional participants mentioned minor abdominal complaints during the test, including pain in epigastrio, flatulence and belching. The individuals reporting abdominal complaints did not show higher I-FABP levels during and post exercise than the individuals without abdominal complaints.

### Exercise-induced changes in liver parameters

**Figure 3:**
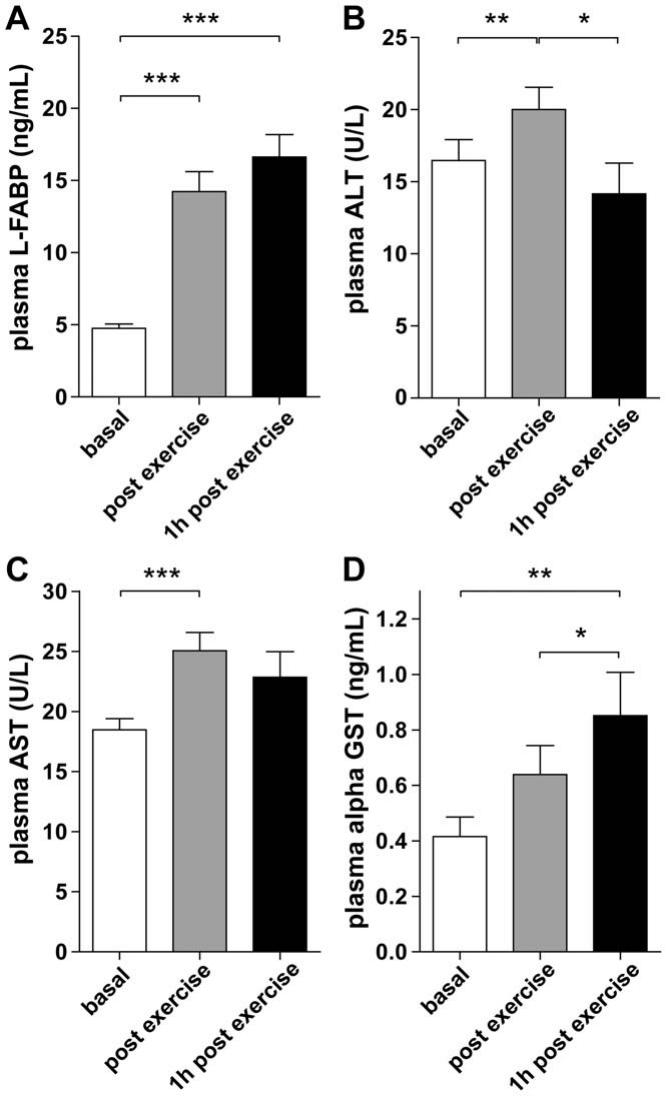
Physical exercise is associated with liver damage. Plasma L-FABP (**A**), ALT (**B**), AST (**C**), and alpha GST (**D**) suggest liver injury after cycling. Data are mean ± SEM (n = 20) of samples collected before, immediately after and 1 hour post cycling (* p<0.05, ** p<0.01, *** p<0.001).

To obtain information on liver injury during exercise, we analyzed a set of four parameters: liver fatty acid binding protein (L-FABP), alanine transaminase (ALT), aspartate transaminase (AST), and alpha-glutathione S-transferase (alpha-GST). Plasma L-FABP levels significantly increased in all individuals upon exercise (4.75±0.30 to 14.2±1.38 ng/ml (p<0.001; Figure 3A)). Plasma ALT and AST levels increased from baseline 16.5±1.40 to 20.0±1.60 U/l (p<0.01; Figure 3B) and 18.5±0.93 to 23.8±0.98 U/l (p<0.001; Figure 3C), respectively. Alpha-GST levels also increased during and after exercise (0.42±0.07 to 0.64±0.10 ng/ml (p = 0.12)), reaching statistical significance at 1 hour post exercise (0.85±0.16 ng/ml (p<0.01 vs. baseline, p<0.05 vs. directly post exercise; Figure 3D). Prolonged release of L-FABP and alpha-GST was observed at least for 1 hour post exercise, whereas plasma ALT and AST levels decreased 1 hour post exercise.

### Exercise-induced changes in renal parameters

While we assumed that exercise caused renal damage, no significant changes in urinary N-acetyl-beta-(D)-glucosaminidase (NAG) levels were observed (p>0.05; Figure 2B). NAG, expressed in tubular epithelial cells [14], which are vulnerable to ischemic injury, is a sensitive marker of renal damage. Our data indicate that exercise, accompanied by enterocyte and hepatocellular injury, does not cause renal damage.

### Exercise-induced GI permeability changes without evidence for bacterial translocation

**Figure 4:**
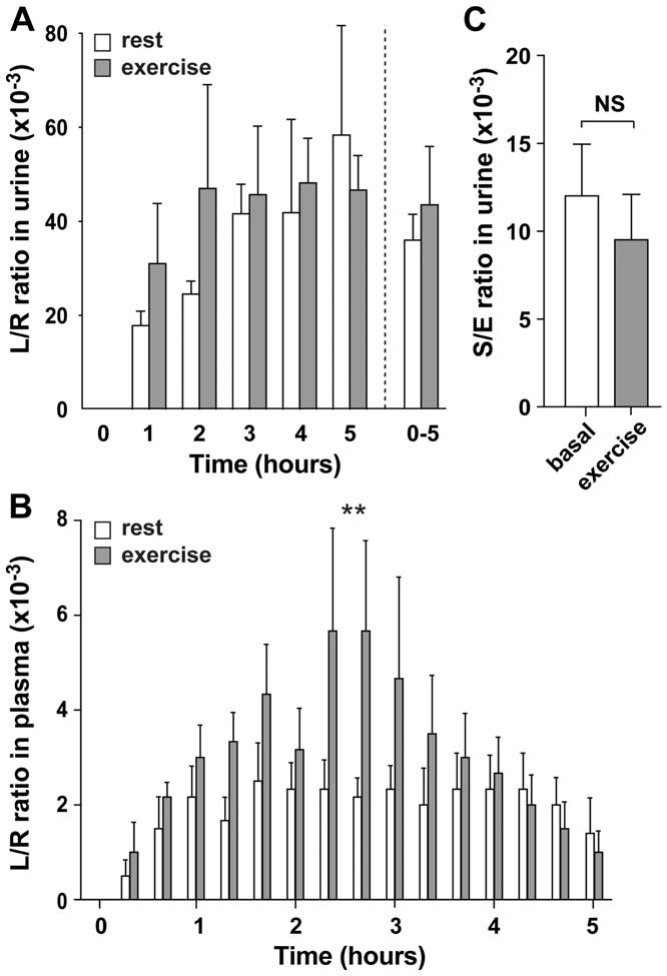
Physical exercise induces a mild increase in small intestinal permeability. **A**) Urinary L/R ratios over time are elevated from baseline, especially in the first 2 hours after cycling, but not to a significant level. **B**) Plasma permeability analysis revealed an overall increase in small intestinal permeability after exercise compared to rest (*P*<0.001). Plasma L/R ratios after exercise at individual time points were not significantly different from L/R ratios in rest. **C**) Permeability of the large intestine, reflected by the 5–24 h urinary S/E ratio, remains unaltered during exercise. Data are mean ± SEM (n = 6). Different from baseline (t = 0) (NS, not significant; ** p<0.001).

To analyze whether the exercise-induced GI hypoperfusion and consequent enterocyte damage resulted in GI permeability changes, we analyzed small intestinal permeability, which was assessed as lactulose/L-rhamnose (L/R) ratio in urine and plasma. All subjects were able to urinate within 10 minutes of the instructed time point, without using Foley catheters. Overall, a trend towards higher small intestinal permeability after exercise was observed in urine, which was especially pronounced in the first two hours of urinary collection (Figure 4A). However, no statistically significant changes were observed in the urinary L/R ratio or the individual sugars after exercise. Since modest, transient changes in intestinal permeability only increase sugar probe excretion transiently, we considered that this increased probe excretion might be overshadowed by normal probe excretion, because all excreted probes accumulate in the bladder before leaving the body. Using our novel approach for sugar probe detection, we could assess the sugar probes in detail by analysis of plasma samples. Plasma analysis revealed an overall increase in small intestinal permeability after exercise compared to rest (p<0.001; Figure 4B). This increased plasma L/R ratio was the result of significantly elevated lactulose concentrations after exercise (p<0.05; data not shown), while plasma rhamnose levels remained unchanged. Interestingly, the plasma L/R ratios correlated significantly with plasma I-FABP levels measured in the same samples during exercise (r_S_: 0.50, p<0.001; data not shown).

Next, correlations between the 0–1 h urinary L/R ratio and the AUC of the plasma L/R ratios determined in the 0–1 h time period were evaluated for similarity, resulting in a R_S_ of 0.42 (p<0.05; data not shown). Finally, the sucralose/erythritol (S/E) ratio was determined in the 5–24 h urinary collections to study large intestinal permeability changes. No changes in large intestinal permeability were observed (Figure 4C).

To assess the translocation of bacterial endotoxin from the intestine into the circulation, EndoCab analysis was performed. The data did not reveal significant changes in endotoxin core antibody levels before and after cycling in the subgroup of participants that underwent permeability analysis (Figure S2A) or in the total group of participants (Figure S2B).

### Exercise-induced inflammatory response

Myeloperoxidase (MPO) and calprotectin were analyzed as parameters of innate inflammation. Plasma MPO levels increased significantly in all individuals upon exercise, from baseline 4.4±0.8 to 33±4.7 ng/ml after cycling (p<0.001; Figure 2C). In line with these results, a significant raise in circulating calprotectin levels was observed (p<0.0001; Figure 2D). Moreover, faecal calprotectin levels increased significantly upon exercise from baseline median 1.07 µg/g (range 0.49–2.20 µg/g) to 1.48 µg/g (range 0.59–40.82 µg/g, p<0.05; data not shown).

## Discussion

The redistribution of blood away from the splanchnic area is an adaptation of the body enabling perfusion of critical tissues in both physiological and pathophysiological situations. During physical exercise, reduction of the splanchnic circulation occurs to meet the increased demand for oxygen and substrates in the active muscle, thereby inducing a physiological state of hypoperfusion in the GI system [8]. This study provides for the first time a comprehensive overview of the processes occurring in response to splanchnic hypoperfusion.

In this study, we demonstrate that in healthy individuals, one hour of physical exercise results in splanchnic hypoperfusion and small intestinal damage, reflected by an increase in plasma I-FABP. Interestingly, the extent of intestinal damage significantly correlated with the exercise-induced hypoperfusion and with mild permeability changes in the small intestine. The most pronounced change in splanchnic perfusion, i.e. the most obvious increase in gap_g-a_pCO_2_, occurred within the first 10 minutes of cycling, which implies that splanchnic perfusion rapidly decreases following the onset of exercise. The speed of adaptation of the splanchnic blood flow is further demonstrated by its fast recovery after cycling, which was also most prominent during the first 10 minutes following cessation of exercise. The rapid decline in splanchnic perfusion during cycling was followed by the development of significant intestinal damage with a time lag of 20 minutes. The data show a correlation between the exercise-induced splanchnic hypoperfusion and enterocyte damage (measured 20 minutes later), which is in line with animal studies and studies performed in critically ill patients demonstrating that intestinal hypoperfusion and ischemia result in intestinal cell damage [4]. The exercise-induced damage to the small intestine was measured using plasma I-FABP and I-BABP, both sensitive plasma parameters of enterocyte damage that are predominantly expressed in the upper half of the villi, where the mature enterocytes reside [15]–[17]. The susceptibility of the mature enterocyte population to low-flow states and ischemic events has been explained by the counter current exchange mechanism in the villus [18], [19], creating a constant low oxygen environment at the villus tip. Intestinal hypoperfusion deteriorates the oxygen-deprived status of the villus tip even more, resulting in rapid loss of epithelial cell membrane integrity and concomitant I-FABP and I-BABP release. The increase in both I-FABP and I-BABP indicates that exercise results in small intestinal damage in general, including the ileum. Interestingly, plasma I-FABP levels gradually decreased in the 60-minute ‘reperfusion’ period. The absence of a second peak in plasma I-FABP levels in this 60-minute period after splanchnic hypoperfusion indicates that detectable reperfusion injury did not occur in our healthy individuals. Our observations are in line with recent work on human intestinal ischemia/reperfusion (I/R) studies from our group showing that after 30 minutes of full intestinal ischemia in an isolated part of the jejunum during a Whipple procedure, plasma I-FABP levels rapidly increase, whereas during reperfusion, a gradual decrease in I-FABP levels was observed [20]. During reperfusion, shedding of the damaged enterocytes prevented the jejunum from a massive I/R-induced inflammatory response [21]. Taken together, these results reflect the ability of the gut to withstand episodes of partial or total deprivation of blood flow, with relatively little damage to the intestinal mucosa.

Having obtained strong indications for reduced splanchnic blood flow, we hypothesized that splanchnic outflow may be reduced during strenuous physical exercise, resulting in hepatocyte damage. Plasma levels of all assessed liver parameters (i.e. L-FABP, ALT, AST, and alpha-GST) elevated significantly after exercise. However, two other factors could be responsible for the phenomenon. First, the increase in these plasma parameters could theoretically be partly attributed to an exercise-induced reduction in plasma volume. The increases, however, largely exceeded the 10% decrease in plasma volume that has been described in athletes in more extreme settings than ours [22]. Alternatively, muscle transaminase release is expected in response to (exercise-induced) muscle injury [23], [24], while L-FABP release could be due to intestinal damage. Therefore, we additionally analyzed plasma alpha-GST, an early marker of hepatocyte injury unaffected by muscle injury, haemolysis, or intestinal injury [25], [26]. In short, the significant rise in the assessed liver parameters after exercise strongly suggests the development of mild hepatocyte damage, which is in line with García-Pagàn et al. who reported a decrease in hepatic blood flow in patients with liver cirrhosis and portal hypertension after only 10 minutes of cycling at submaximal workload [27].

In contrast to the intestinal and liver data, we did not observe indications for renal damage during exercise. At this stage, it is unclear whether there was no hypoperfusion in the kidneys during cycling, or whether the kidney is more resistant to physiological hypoperfusion. Previous studies support the former, showing relatively unchanged renal flow after short-term splanchnic blood redistribution [28], [29].

In addition to the observed epithelial cell integrity loss, activation of the inflammatory response was shown. Inflammation is characterized by neutrophil influx and activation, resulting in the release of MPO and calprotectin, as seen in the present study. MPO plays a role in oxidative processes, while calprotectin and one of its constituents, myeloid-related protein-8 (Mrp8, S100A8), are identified as strong activators of the innate Toll-like receptor 4 signaling pathway, resulting in the expression of pro-inflammatory cytokines [30]. In addition to a rise in plasma calprotectin levels, increased faecal calprotectin concentrations were found after cycling in our healthy individuals, reflecting sequestration and activation of neutrophils in the intestinal mucosa, suggesting the gut to be an additional source of inflammatory products after physical exercise. Inflammation and neutrophil influx have been described previously in the human intestine, as part of I/R injury [31], while a study by Mortensen et al., assessing femoral arterial-venous differences during knee extensor exercise, pointed towards release of calprotectin from muscle tissue during exercise [32]. We consider the release of both calprotectin and MPO to be enhanced in sites of tissue injury and inflammation, including muscle tissue (during exercise), but also the gut mucosa. Loss of gut barrier function, due to cellular injury and inflammation, could result in bacterial translocation. Indeed, Jeukendrup et al. found mild endotoxaemia (5–15 pg/ml) and increased levels of C-reactive protein in the majority of participants after finishing an approximately 12 h long-distance triathlon [33]. While our data did not point towards bacterial translocation, the existence of mucosal damage and inflammation in the GI tract did suggest loss of epithelial barrier function in our healthy volunteers. In line, analysis of the sugar probes in plasma using our novel HPLC/MS approach revealed increased small intestinal permeability, while these permeability changes correlated with the observed intestinal injury. Interestingly, the classical analysis of sugar probes in urine did not reveal significant changes in permeability, which might be explained by the mild and temporary nature of the increase in small intestinal permeability. In short, the absence of endotoxaemia and the mild changes in small intestinal permeability found in the current study underline the physiological basis of the exercise-induced changes in GI homeostasis.

In the current study, exercise did not significantly increase large intestinal permeability. It could be argued that the large intestine is better protected against episodes of hypoperfusion, or that blood flow in the large intestine is better maintained to prevent the translocation of bacteria and their products from the lumen to the circulation. The latter is suggested by a study of Murray et al. that found a decrease in rectal mucosal blood flow of almost 30% after acute physical stress [34], while decreases ranging from 43 to approximately 80% have been reported for splanchnic blood flow during physical exercise [8], [35].

In this study, we show the development of intestinal damage due to physiological hypoperfusion. Our data imply that the healthy human small intestine can withstand transient hypoperfusion, since the observed enterocyte injury did not produce GI symptoms in our healthy individuals. However, prolonged episodes of hypoperfusion may cause more severe epithelial injury that may give rise to abdominal symptoms, as observed in endurance trained athletes [36]. Furthermore, it is tempting to speculate that in individuals with hampered vascular function or decreased cardiac output, physiological conditions such as ‘regular’ physical exercise and other daily activities, will rapidly result in splanchnic hypoperfusion, ultimately leading to enterocyte damage and increased gut permeability. In patients with compromised vascular function, hypoperfusion-induced epithelial dysfunction may give rise to endotoxaemia, thereby enhancing their state of chronic inflammation, as suggested by the group of Anker for patients with chronic heart failure [6], [37]. We assume that in addition to such patients, also relatively healthy elderly experience episodes of splanchnic hypoperfusion during daily activities due to the ageing of the splanchnic vascular system.

In conclusion, our study demonstrates that a single, 60-minute bout of moderate-to-high intensity exercise is accompanied by splanchnic hypoperfusion and transient epithelial integrity loss in healthy young males, reflecting the ability of the gut to withstand to episodes of physiological stress with minor consequences.

## Methods and Materials

### Ethical approval

This study was approved by the medical ethical committee of Maastricht University Medical Centre+, and conducted in accordance with the Declaration of Helsinki (revised version, October 2008, Seoul).

### Participant characteristics

Healthy male volunteers were recruited via posters at the University. All volunteers spent 4 to 10 hours per week performing endurance sports as part of their normal life style. The volunteers had no abdominal complaints during daily activities, had not taken any medication for at least one month prior to participation, had no history of GI disease, and had had no abdominal surgery. Volunteers were informed about the nature and risks of the experiments. Written consent was obtained 5 days before the experiments.

Participants had a mean age of 23.6±0.7 years and body mass index of 21.0±0.4 kg/m^2^. Subjects' baseline characteristics and maximum workload capacity did not differ between the study groups (Table 1). All participants were able to complete the 1-hour exercise bout, except for 1 volunteer, who discontinued cycling after 42 minutes due to exhaustion.

**Table 1:** Baseline characteristics of the healthy male participants.

	GI perfusion	Intestinal damage	GI permeability	p-value
Group size (no. of participants)^1^	9	15	6	
Age (years)	24.2±1.0	23.8±0.8	25.0±0.6	0.67
Height (m)	1.79±0.02	1.80±0.02	1.79±0.00	0.79
Weight (kg)	68.4±2.8	68.1±1.7	66.8±3.8	0.92
Body Mass Index (kg/m^2^)	20.3±0.6	21.0±0.5	20.8±0.7	0.68
Total body: fat percentage (%)	11.6±0.7	11.9±0.8	12.5±0.6	0.81
fat mass (kg)	8.0±0.6	8.1±0.6	8.4±5.8	0.92
fat free mass (kg)	61.1±2.6	60.3±1.5	59.4±3.8	0.91
Both legs: fat percentage (%)	12.6±0.8	12.6±0.9	13.5±0.6	0.77
fat mass (kg)	3.1±0.3	3.1±0.3	3.3±0.3	0.87
lean mass (kg)	20.2±1.2	20.5±0.7	20.1±1.5	0.97
Maximum workload (W/kg)	5.0±0.1	5.2±0.1	5.3±0.1	0.33

Data are presented as mean ± SEM.

1 Of all study participants, three subjects participated in all three substudies, and four subjects participated in two substudies. Hence, in total, 20 individuals took part in the study.

### Pre-exercise restrictions/arrangements

Prior to the experiments, maximal workload capacity was assessed on a stationary cycle ergometer (Lode Excalibur, Groningen, the Netherlands), during electrocardiographic monitoring (MAC 5500, GE Medical Systems, Freiburg, Germany) to exclude cardiologic abnormalities. A dual energy X-ray absorptiometry scan (DXA™, Hologic Inc., the Netherlands) was performed to assess body composition.

Test subjects recorded dietary intake 2 days prior to the first experimental day, and maintained dietary intake as recorded for subsequent test days to prevent dietary influence. Participants were not allowed to consume alcohol or caffeine 2 days prior to each test day. Moreover, participants maintained normal activities of daily living, but refrained from strenuous physical activity. The evening before each test day, participants received a standardized meal (1.7 MJ, consisting of 62.6 g carbohydrate, 18.9 g protein and 7.9 g fat).

### Study design and sampling

Subjects were tested after an overnight fast. The test design is depicted as Figure S1. In short, a catheter (20 Gauge, Braun, Melsungen, Germany) was placed in the participant's forearm vein. Every 10 minutes, blood was collected into pre-chilled EDTA tubes (Vacucontainer, Becton Dickinson (BD), Helsingborg, Sweden), and kept on ice. Urine was collected by the participants in 1 l plastic cups before and within 1 hour after exercise. Urinary volume was recorded and 4 ml was directly transferred to pre-chilled tubes. Blood and urine samples were centrifuged within 1 hour at 4°C at 2300×*g* for 15 minutes and immediately stored at −80°C until analysis. Following 1 hour rest in supine position, participants started cycling at 150 W. After 3 minutes, workload was increased to 70% of the individual's pre-assessed maximal workload capacity. Subjects maintained pedal rates of 60 rpm, and workload was decreased by 25 W if the participant was unable to maintain 60 rpm. Participants were only allowed to drink a maximum of 200 ml tap water per 10 minutes. Faeces were collected by 6 participants, before and after cycling, and stored at −4°C until analysis. Subjects were asked to report GI complaints experienced on the test day and the following day.

### Assessment of GI perfusion

Gastric air tonometry was performed in 9 participants. The evening before and 1 hour prior to the experiment, participants ingested 150 mg ranitidine orally (GlaxoSmithKline, Zeist, the Netherlands) to suppress gastric acid production, which interferes with carbon dioxide, thereby affecting tonometry results [38]. A nasogastric Tonometrics catheter (8 French, Datex Ohmeda, Finland) was introduced and fixed to both nasal flares. Gastric pCO_2_ was measured at 10-minute intervals using an automated capnograph (Tonocap TC-200, Datex Ohmeda, Finland). To avoid the use of an arterial catheter, an intravenous catheter (22 Gauge, BD) was placed in a dorsal hand vein and the hand was placed in a hot box heated to 60°C, to arterialize the blood for measuring arterial pCO_2_ [39], using a Radiometer ABL 510 (Copenhagen, Denmark). Blood samples were collected simultaneously with the tonometry measurements and gap_g-a_ pCO_2_ was calculated.

### Assessment of intestinal damage

I-FABP, a sensitive marker of intestinal cell damage, was used to determine small intestinal damage [15]. Plasma I-FABP was determined using an ELISA (Hycult Biotechnology, Uden, the Netherlands; detection window 20–5,000 pg/ml) according to the manufacturer's instructions in samples taken before (t = 0), during (t = 10–50 minutes) and after (t = 60–120 minutes) cycling. Plasma ileal bile acid binding protein (I-BABP), a sensitive damage marker of ileal epithelium, was measured before (t = 0) and after (t = 60 minutes) cycling, using an in-house developed ELISA as previously described (detection window 0.32–5.0 ng/ml) [16].

Plasma markers for tissue damage and inflammation were analyzed from every subject who completed the 1-hour exercise bout, irrespective of the substudy in which he participated (Figure S1). Some subjects participated in more than one substudy, leaving 20 unique study subjects for these analyses, unless stated otherwise.

### Assessment of liver damage

Liver injury was assessed by L-FABP, ALT, and AST. Plasma L-FABP is a sensitive indicator of hepatocellular injury [13]. However, as expression of L-FABP has been described in renal and intestinal epithelium [40], this marker was combined with the classically used parameters ALT and AST. In addition, we determined plasma levels of alpha-GST, which is advocated to be an early and more specific marker of liver injury and unaffected by muscle injury or haemolysis [25], [26]. All liver markers were determined before, immediately after, and 1 hour after cessation of cycling. Plasma L-FABP was measured using a commercial ELISA according to the manufacturer's instructions (Hycult, detection window 0.20–25 ng/ml). Plasma ALT and AST were determined by routine enzymatic assays at the Clinical Chemistry Laboratory. Plasma alpha-GST levels were assessed using ELISA, as described previously [41].

### Assessment of kidney damage

NAG was determined using an enzyme colorimetric assay according to the manufacturer's instructions (HaemoScan, Groningen, the Netherlands). Concentrations were normalized to urinary creatinine values, measured by routine assays at the Clinical Chemistry Laboratory and expressed as Units/mmol creatinine.

### Assessment of GI permeability

GI permeability was determined using a mix of 1 g lactulose (Centrafarm, Etten-Leur, the Netherlands), 1 g sucralose (Brenntag, Sittard, the Netherlands), 1 g erythritol (Danisco, Copenhagen, Denmark), 1 g sucrose (Van Gilse, Dinteloord, the Netherlands), and 0.5 g L-rhamnose (Danisco) dissolved in 150 ml tap water, in a subset of 6 participants.

The study design, depicted in Figure S1, consisted of 2 test days, one to test basal permeability and one for exercise-induced permeability. Participants fasted overnight. The sugar bolus was ingested after 30 minutes of rest in supine position, or after 30 minutes of cycling. Blood was sampled every 20 minutes and urine every hour, up to 5 hours post ingestion. All subjects urinated in plastic cups within 10 minutes of the instructed time point. In addition, subjects collected their 5–24 hour urine at home and returned the provided bottles the next morning, when urine was immediately processed.

Combined HPLC (Model PU-1980 pump, Jasco Benelux, Maarsen, the Netherlands) and mass spectrometry (Model LTQ-XL, Thermo Electron, Breda, the Netherlands) were used to determine urinary and plasma sugar concentrations. Lactulose, rhamnose, and L/R ratio were determined in urinary samples to assess small intestinal permeability. Since physiological, transient permeability changes were expected, and our novel permeability assay allowed the detection of sugar probes in plasma, we also determined lactulose and rhamnose concentrations, and L/R ratios in plasma. Both lactulose and rhamnose are degraded in the colon. Therefore, the ratio between two inert sugar probes, sucralose and erythritol, was determined in the 5–24 h urine to assess large intestinal permeability. Since we did not draw blood in this time period, we did not perform large intestinal permeability analysis in plasma.

### Assessment of inflammatory activation products

Circulating myeloperoxidase (MPO) and calprotectin, antimicrobial proteins that are excreted from neutrophils upon activation, are considered early parameters of systemic inflammatory activation [42], [43]. Active MPO is known to elicit secretion of other cytokines including tumor necrosis factor-α and interleukins [44], which were therefore less useful. MPO was measured in plasma before and after cycling using a commercial ELISA (Hycult; detection window 0.40–100 ng/ml). Since evidence is accumulating that inflammatory products are produced in contracting and skeletal muscle [32], [45], we determined both circulating and faecal calprotectin. The latter is considered a strong indicator of intestinal inflammation in specific, and has been found to correlate to other parameters of intestinal inflammation in inflammatory bowel disease [46] and enteropathy induced by non-steroidal anti-inflammatory drugs (NSAID) [47]. Calprotectin was measured before and after cycling, using a commercial calprotectin ELISA (standard 1.6–100 ng/ml). Faeces were collected by the participants in provided faecal sampling cups. Time of collection was reported by the participants. All faecal samples were collected within 7 hours after cycling. For faecal analysis, faeces were thawed, and 100 mg was added to 4.9 ml of extraction buffer (0.1 M Tris, 0.15 M NaCl, 1.0 M urea, 10 mM CaCl_2_ 2H_2_O, 0.1 M citric acid, 0.5% BSA, pH 8.0) was added [48]. After 30 minutes shaking, 1 ml of suspension was centrifuged at 10,000 rpm for 20 minutes at 4°C. Faecal calprotectin concentrations were measured in supernatant using the calprotectin ELISA.

Endotoxin core antibodies in plasma were determined using EndoCab ELISA (Hycult Biotechnology, Uden, the Netherlands; detection window 0.13–8.0 IgG median units (GMU)/mL) according to the manufacturer's instructions in samples taken before and after cycling.

### Statistical analysis

Statistical analysis was performed using GraphPad Prism (Version 5.00, GraphPad Software for Windows, San Diego California, USA). Normality of all data was verified by the Kolmogorov-Smirnov test. All normally distributed data are presented as mean ± standard error of the mean (SEM), not normally distributed data as median and range. Outliers identified using Grubbs analysis were excluded from statistical analysis. Continuous data were analyzed using repeated measures analysis of variance with Tukey's or Dunnett's post-hoc test for multiple comparisons. Basal and post exercise values were compared using the non-parametric Wilcoxon signed rank test, considering the small number of study participants. Within-person correlations between gap_g-a_pCO_2_ and plasma I-FABP, and plasma I-FABP and plasma L/R ratio were computed by normalizing both data sets, which enables the assessment of the pure association of both variables by calculating the Spearman correlation coefficient r_S_. Linear regression was used to visualize the correlation. A p<0.05 was considered statistically significant.

## Supporting Information

Figure S1 **Time frame of GI perfusion, intestinal damage and GI permeability study.**
(PDF)

Figure S2 **One hour of strenuous physical exercise does not lead to bacterial translocation.** **A**) Plasma IgG levels against endotoxins did not change significantly upon physical exercise in the subgroup of six participants that underwent permeability analysis. **B**) Plasma analysis for IgG against endotoxins in the total group of participants did not reveal significant changes. Data are mean ± SEM. Different from baseline (t = 0) (NS, not significant).
(PDF)
