# Supplementary material for: Exercise-Induced Splanchnic Hypoperfusion Results in Gut Dysfunction in Healthy Men
Source: PLoS One. 2011 Jul 21;6(7):e22366. doi: 10.1371/journal.pone.0022366 (PMC3141050; doi:10.1371/journal.pone.0022366)
Supplement: Figure S1 — Time frame of GI perfusion, intestinal damage and GI permeability study. (PDF) [file pone.0022366.s001.pdf]

Supporting Information

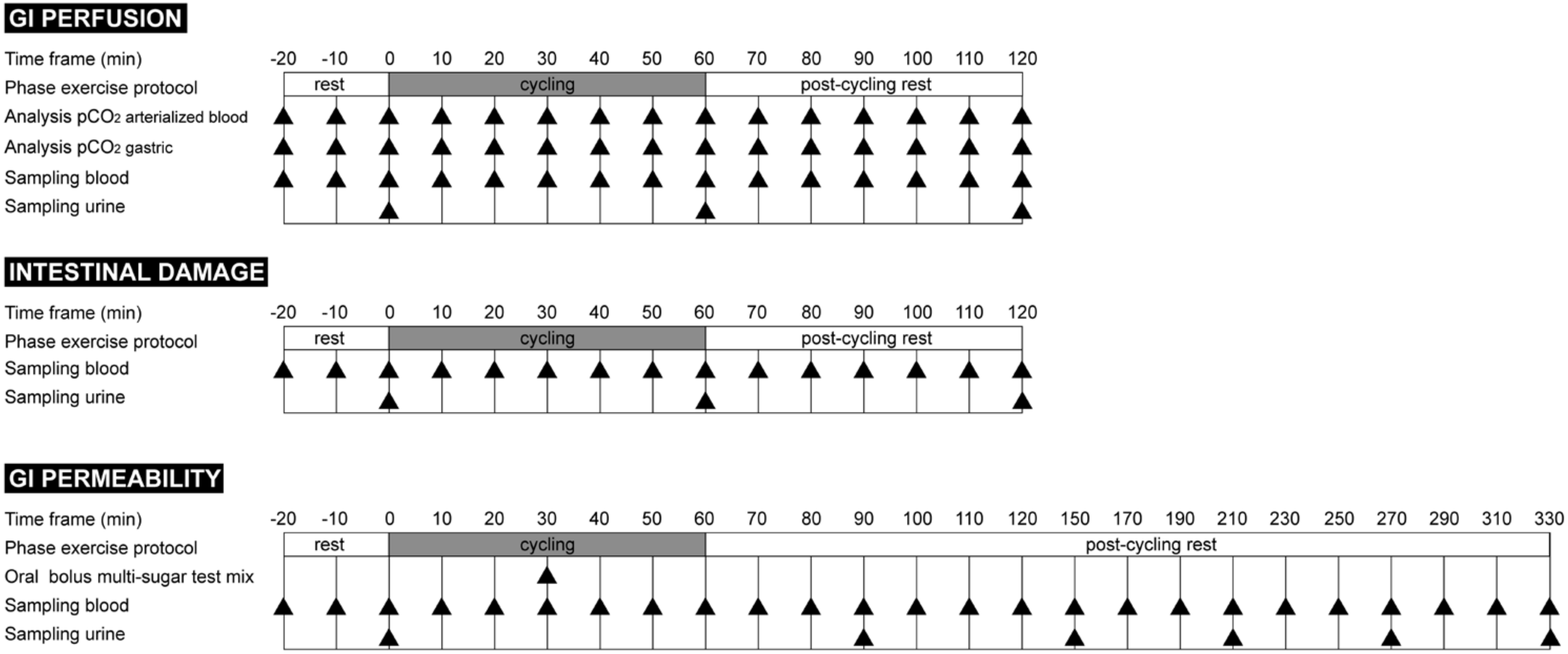

Figure S1. Time frame of GI perfusion, intestinal damage and GI permeability study.
