# Supplementary material for: Exercise-Induced Splanchnic Hypoperfusion Results in Gut Dysfunction in Healthy Men
Source: PLoS One. 2011 Jul 21;6(7):e22366. doi: 10.1371/journal.pone.0022366 (PMC3141050; doi:10.1371/journal.pone.0022366)
Supplement: Figure S2 — One hour of strenuous physical exercise does not lead to bacterial translocation. A) Plasma IgG levels against endotoxins did not change significantly upon physical exercise in the subgroup of six participants that underwent permeability analysis. B) Plasma analysis for IgG against endotoxins in the total group of participants did not reveal significant changes. Data are mean ± SEM. Different from baseline (t = 0) (NS, not significant). (PDF) [file pone.0022366.s002.pdf]

Supporting Information

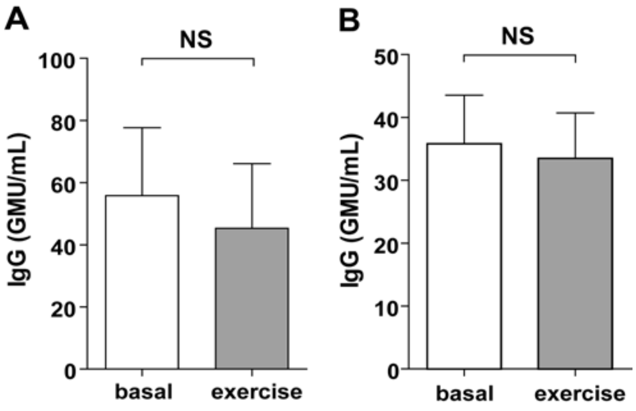

Figure S2. One hour of strenuous physical exercise does not lead to bacterial translocation.
A) Plasma IgG levels against endotoxins did not change significantly upon physical exercise in the subgroup of six participants that underwent permeability analysis. **B)** Plasma analysis for IgG against endotoxins in the total group of participants did not reveal significant changes. Data are mean ± SEM. Different from baseline (t = 0) (NS, not significant).
